# Genetic characterization of S1 gene of infectious bronchitis virus isolated from commercial poultry flocks in West Java, Indonesia

**DOI:** 10.14202/vetworld.2019.231-235

**Published:** 2019-02-11

**Authors:** Rahajeng Setiawaty, Retno Damajanti Soejoedono, Okti Nadia Poetri

**Affiliations:** 1 National Veterinary Drug Assay Laboratory, Jl Raya Pembangunan, Gunung Sindur, Bogor 16340, Indonesia; 2 Department of Animal Diseases and Veterinary Public Health, Faculty of Veterinary Medicine, Bogor Agricultural University, Jl. Agatis, Kampus IPB, Dramaga, Bogor 16680, Indonesia

**Keywords:** genetic characterization, Indonesia, infectious bronchitis virus, poultry, S1 gene, West Java

## Abstract

**Background and Aim:**

Infectious bronchitis (IB) is still a major problem among poultry industry in Indonesia, IB outbreaks continue to happen even in vaccinated flocks. The emergence of new IB virus (IBV) variants might lead to mismatching between vaccine virus strain and circulating virus strain, this may be a reason of vaccination failure. Information about circulating IBV in a region is important to decide which IB vaccine should be used. However, information about recent IBV strains which circulated in Indonesia and their genetic characters were limited; therefore, the aim of our research was to determine the genetic characterization of S1 gene of IBV isolated from commercial poultry flocks in West Java, Indonesia.

**Materials and Methods:**

A total of 47 viral isolate samples collected from chickens with clinical sign and reduced in egg production. Six IB live vaccines were used as control, the reference vaccines represent IBV strains including H120, H52, 4/91, CR88, 233A, and 1-96. Primers XCe2+ and XCe2- were used to amplify S1 gene partially.

**Results:**

Twenty-six of 47 samples showed positive result to S1 gene of IBV by reverse transcription-polymerase chain reaction. Three IBV isolates, Indonesia/K233A31/18, Indonesia/K4A9/17, and Indonesia/P3/17, were selected for nucleotide sequencing. Phylogenetic analysis of 352 nucleotides of the partial S1 gene shows that isolates Indonesia/K4A9/17 and Indonesia/K233A31/18 have 100% homology with IBV vaccine strain 4/91, while isolate Indonesia/P3/17 has 100% homology with IBV vaccine strain 233A.

**Conclusion:**

Our result indicates that at least two IBV strains were circulating among poultry in West Java, Indonesia, which is IBV close to vaccine strain 4/91 and 233A. The present study provides updates on the circulating IBV in commercial poultry flocks in West Java, Indonesia, and might use as guidance on selecting a proper IB vaccine strain to improve IB vaccination efficacy in certain region.

## Introduction

Infectious bronchitis (IB) is a poultry disease caused by *Coronavirus* of *Nidovirales* order [1]. *Coronavirus* is enveloped, non-segmented, single-stranded, and RNA’s positive sense, comprises approximately 27–32 Kb in size [2]. All *Coronaviruses* have four structural proteins, glycoprotein spike, matrix, nucleoprotein, and envelope consist of lipid bilayer and three glycoprotein M, S, and HE [3]. The S protein has two glycopolypeptide components which are S1 and S2. The spike protein S1 undergoes inhibitor of agglutination and induces neutralizing antibody [2]. S1 protein functioned as differentiating factor among IB virus (IBV) strains and as a main target of genotype characterization. It also plays an important role in attachment and virus entries into cells through cyanic acid receptor [4]. Amino acid variation in glycoprotein S1 took important place to tissue tropism and IB virulence [5].

IB is still a serious problem among poultry industry in Indonesia, the prevalence of the disease is 40–60% in Java Island [6]. Outbreaks were also occurred at vaccinated flocks, indicating vaccination failure; however, vaccination is the only practical means of controlling IB. Problem in vaccination is that it is only partially successful due to the continual emergence of antigenic variants. IBV strains within a geographic region are unique, even many countries share same antigenic types, so the selection of an appropriate antigenic type for the region is important, given the existence of wide antigenic variation [7]. The variants of IBV have not been well-documented in Indonesia since the lack of the characterization of this virus [8]. Understanding epidemiological condition and virus changes are important in designing IB vaccination strategies, to provide greater protection against enzootic strains, and the vaccination must be commonly practiced based on the field needs [9].

The previous study showed that the majority of IBV strain isolated in Indonesia were related to Massachusetts (Mass) and Connecticut (Conn), and serotype N2/62 originated from Australia [10], IBV local isolates [11], IBV close to vaccine virus strain 4/91 [12], and IBV originated from Taiwan and China [8].

However, limited information available about IBV strain circulating among poultry in Indonesia and its genetic character; therefore, the aim of our study was to determine IBV field strain and genetic characterization of S1 gene of IBV isolated from poultry in West Java, Indonesia, to provide an update on cocirculating IBV variants in this region.

## Materials and Methods

### Ethical approval

The present study was performed in accordance with the regulations for Research in Animal Health of Indonesian Law on Livestock and Animal Health (UU/18/2009, article 80).

### Samples

A total of 47 samples isolated from problematic flocks showing IB such as clinical symptoms and reduction in production were used in this study. The samples were collected from commercial poultry flocks in some district in West Java Province: Sukabumi (n=36), Cianjur (n=1), Tasikmalaya (n=4), Bogor (n=4), and Subang (n=2). The samples were organ, cloacal swab, and tracheal swab. Six IB live vaccines were used as positive control, the vaccine represents IBV strain H120, H52, 4/91, CR88, 233A, and 1–96.

### Viral RNA extraction

Viral RNA was extracted using the total RNA Mini Kit (Geneaid®), extraction procedure was based on manufacturer’s instructions. The RNAs were dissolved in 50 µl RNase-free water and directly used for subsequent reverse transcription-polymerase chain reaction (RT-PCR) or stored at −20°C.

### Primers for amplification

Partial S1 gene amplification using one pair of primer [13]: Forward XCE2+5’−CAC TGG TAA TTT TTC AGA TGG−3’ and reverse XCE2−5’−CCTC TAT AAA CAC CCT TACA−3’, the expected PCR product size is 466 bp.

### Amplification

The PCR amplification reaction was carried out in 50 µl containing 5 μl RNA template, 25 μl 2× MyTaq one step mix (1×), 0.5 μl RT, 1 μl Ribosafe RNAse inhibitor, DEPC-H_2_O (MyTaq™ OneStep RT-PCR Kit) up to 45 µl, and 4 μl primer (400 nM). Amplification reaction was carried out with thermal profile at 45°C, 20 min RT 95°C, 1 min polymerase activation, then 40 cycles (denaturation 94°C, 15 s; annealing 55°C, 30 s; and extension 72°C, 30 s) followed by final extension 72°C, 7 min.

### Nucleotide sequencing

About 30 µl each of RT-PCR product of amplified S1 sequenced using BigDye® Terminator version 3.1 Cycle Sequencing Kit (Thermo Fisher Scientific, USA) with forward XCE2+ and reverse XCE2−primer. These samples were sent for sequencing (First Base, Malaysia) and sequenced from both directions. The sample nucleotide sequence results are compiled, aligned, and compared with sequence of S1 gene of control positive and also with sequence data of S1 gene of published IBV available in the GenBank (Table-1). Phylogenetic trees were constructed using maximum likelihood of the general time reversible model of MEGA 7 software (http://www.megasoftware.net) [14,15].

**Table-1:** Nucleotide and amino acid identity of selected IBV isolates and vaccine strains.

Samples	1	2	3	4	5	6	7	8	9
	Amino acid identity								
H120		0.25	0.27	0.28	0.28	0.26	0.26	0.26	0.28
4/91	0.26		0.07	0.07	0.08	0.10	0.01	0.01	0.07
CR288	0.28	0.03		0.04	0.10	0.10	0.06	0.06	0.04
233A	0.27	0.03	0.02		0.08	0.06	0.07	0.07	0.00
1/96	0.27	0.05	0.06	0.05		0.10	0.09	0.09	0.08
H52	0.23	0.05	0.05	0.03	0.07		0.11	0.11	0.06
Indonesia/K233A31/18	0.26	0.00	0.03	0.03	0.05	0.05		0.00	0.07
Indonesia/K4A9/17	0.26	0.00	0.03	0.03	0.05	0.05	0.00		0.07
Indonesia/P3/17	0.27	0.03	0.02	0.00	0.05	0.03	0.03	0.03	

Nucleotide identity, IBV=Infectious bronchitis virus

## Results

Twenty-six of 47 samples showed positive result to S1 gene of IBV by RT-PCR. These samples were isolated from three different farms in West Java; therefore, we choose three positive samples only, one viral isolate from each farm for further nucleotide sequencing analysis. These three isolates are Indonesia/K4A9/17 and Indonesia/P3/17 from Sukabumi, while Indonesia/K233A31/18 from Bogor.

**Figure-1:**
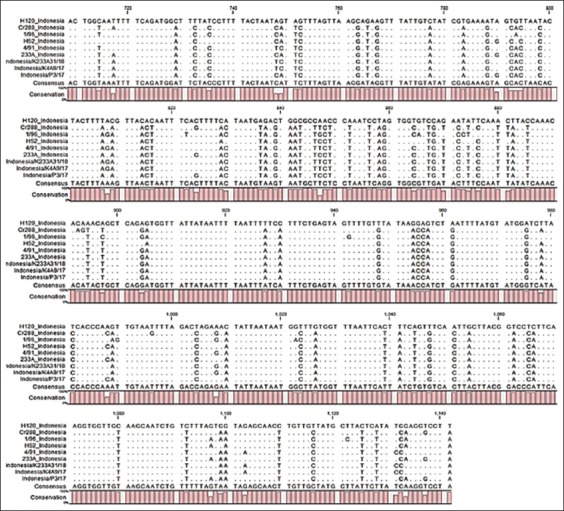
Partial sequence of the S1 gene of infectious bronchitis virus isolates and vaccine strain.

Nucleotide sequencing of 352 nucleotide partial gene S1 indicates that isolates Indonesia/K4A9/17 and Indonesia/K233A31/18 have 100% homology with IBV vaccine strain 4/91, while isolate Indonesia/P3/17 has 100% homology with IBV vaccine strain 233 A. Among these three IBV isolates, the deviation between Indonesia/K4A9/17 and Indonesia/K233A31/18 was 0%, while Indonesia/K4A9/17 and Indonesia/K233A31/18 deviate 0% to Indonesia/P3/17. Difference of S1 gene sequence between these three IBV isolates with other vaccine strain was 2–29% (Table-1). There was no deletion or insertion occurred inside the S1 genes. Point mutation throughout nucleotide sequences was not detected either (Figure-1).

There are 11 nucleotide differences within three IBV isolates (Table-1). Differences between three samples located in nucleotides: 749, 790, 808, 824, 988, 1008, 1097, 1103, 1131, 1133, and 1137. There are no changes in amino acid residue - 304 that correlated with nucleotide - 894. There are no changes in nucleotide order A-ACA-G from cytosine to timina and amino acid histidine from the three samples or vaccine strain. XCE primers used in this study only able to amplify third quarter part of hypervariable regions 3 (HVRs 3) in S1 gene, whereas HVRs 3 located at amino acid residue 274–387. Amino acid residue indicates no deletion, insertion, or mutation at position 298 (Histidine) (Figure-1).

**Figure-2:**
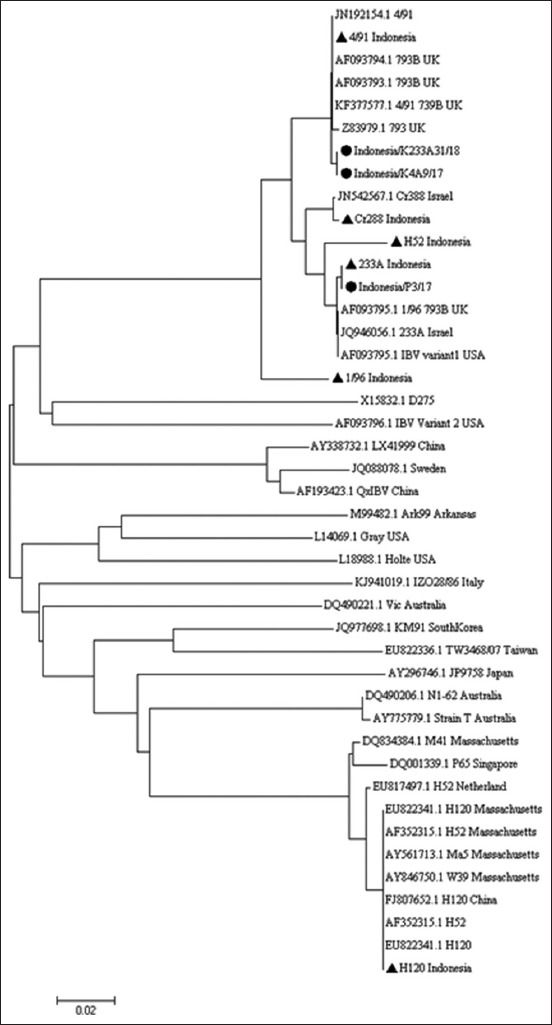
Phylogenetic tree of selected infectious bronchitis virus (IBV) isolates, vaccine virus, and other IBV from GenBank. IBV Indonesia/K23331A/18, Indonesia/K4A9/17, and Indonesia/P3/17 (●), vaccine virus (▲), and IBV from GenBank.

The phylogenetic tree (Figure-2) of the aligned nucleotide of partial S1 gene was constructed using maximum likelihood of the general time reversible model of MEGA 7 (www.megasoftware.net) [14,15], the tree showed a close relatedness of two IBV isolates (Indonesia/K4A9/17 and Indonesia/K233A31/18) with reference IBV strain 793 (Accession No. Z83979), strain 793B (Accession No. KF377577), strain 793B (Accession No. AF093793), strain 793B (Accession No. AF093794), and strain 491 (Accession No. JN1921541) isolated in the United Kingdom (UK), while other isolate Indonesia/P3/17 has close relatedness to reference IBV strain 233A (Accession No. JQ946056), Variant 1 (Accession No. AF093795), and strain 1/96 793B (Accession No. AF093795) isolated from Israel, the United States of America (USA), and the UK, respectively. Based on genetic characterization on S1 gene sites, isolates Indonesia/K4A9/17 and Indonesia/K233A31/18 were likely belonged to IBV close to vaccine strain 4/91, and isolate Indonesia/P3/17 likely belonged to IBV close to vaccine strain 233A.

## Discussion

Ronoharjo, in 1977, was the first who isolated IBV in Indonesia, a few years later Darminto in 1985 were able to isolated IBV [8]. These viruses belonged to four serotypes group: One group close to IBV Mass, another close to IBV Conn, and the other two groups close to IBV from Australia [10]. The emergence of IBV variants in Indonesia was first detected by Dharmayanti *et al*. [16], and later on, the study of IBV variants was conducted by Dharmayanti and Indriani [8] which detected IBV variants close to Taiwan and China strain. IBV has a high capacity for genetic change through mutation, and these genetic mechanisms might lead to emergence of new IBV serotypes and variants and evolution of IBV also influenced by application of multiple vaccine strain [17-19]. Therefore, updating epidemiological status of IBV through genetic characterization is important for designing appropriate vaccination program in a certain region.

The spike protein or S1 subunit of S glycoprotein is the major determinant of IBV so that a minor change in amino acid sequence of this protein would transform the virus strain [20]. Changes in amino acid of S1 gene >5% may result in reducing the ability of a vaccine to protect an individual from field virus attack [21]. Determinant region for IB serotyping was located in the first 395 amino acid of S1 gene. Amino acid epitopes play an important role in virus critical antigenic were located at position 296 (amino acid Glutamine, Threonine and Alanine replaced by Histidine, Threonine and Alanine or Serine, Threonine and Alanine) and position 378 (amino acid Proline, Arginine and Glycine replaced by Proline Arginine Leucine, these substitutions cause decreasing of vaccine protection and produced new IBV strain. Variation of nucleotides and amino acids may lead to serotype changes, which, in turn, changes tissue tropism and pathogenicity of the virus [22].

Based on our partial sequence of S1 gene results, there are no substitutions in 894-nt and amino acid residue - 298. Partial nucleotide sequencing of S1 gene of IBV Indonesia/K4A9/17 and IBV Indonesia/K233A31/18 was 100% identical with vaccine strain 4/91, and IBV Indonesia/P3/17 was 100% identical to vaccine strain 233A. No mutation, insertion, or deletion detected in nucleotide sequence of IBV isolates in this study. An explanation could be that, this is resulted from reisolation of vaccine strain virus circulating in the susceptible host or there is high recombination of IBV genome during cocirculation of vaccine virus and field virus [18,19].

Phylogenetic three showed that three IBV isolates were relatively close to IBV from the UK, Israel, and the USA, respectively. In Indonesia, a latest study about genetic characterization of IBV isolated by Dharmayanti and Indriani [8] showed that two cocirculating IBV IB/WJ.2010 and IB/JB.1990 were close to IBV originating from China, while other IBV IB L8.1996 was close to IBV originating from Taiwan, and all of these strain did not show proximity to vaccine strain M41, H120, and Conn which is widely used in Indonesia. The present study indicates that during these years, IBV circulating in Indonesia has been evolved. The continuous existence of IBV variant in the field shows the importance of regular surveillance to get an update on epidemiological situation of IB infection and emerges of new IBV variants to improve efficacy of IBV control strategies in certain country.

## Conclusion

The present study provides updates on the circulating IBV in commercial poultry flocks in West Java, Indonesia. Partial sequencing of the S1 gene revealed at least two IBV strains circulating among poultry in West Java, which is IBV close to vaccine strain 4/91 and IBV close to strain 233A. Based on phylogenetic three, these three isolates were relatively close to IBV from the UK, Israel, and the USA, respectively.

## Authors’ Contributions

RS performed the work designed by RDS and ONP. All authors contributed in writing and revision of the manuscript. All authors read and approved the final manuscript.
